# Application of a novel prognostic invasive lesion index in ductal carcinoma in situ with minimal invasion of the breast

**DOI:** 10.1002/cam4.1175

**Published:** 2017-10-04

**Authors:** Xiaofang He, Feng Ye, Mei Li, Ping Yu, Xiangsheng Xiao, Hailin Tang, Xiaoming Xie

**Affiliations:** ^1^ Department of Breast Oncology Sun Yat‐sen University Cancer Center State Key Laboratory of Oncology in South China Collaborative Innovation Center for Cancer Medicine Guangzhou Guangdong China; ^2^ Department of Pathology Sun Yat‐sen University Cancer Center Guangzhou Guangdong China

**Keywords:** Ductal carcinoma in situ with minimal invasion of the breast, lymph node metastasis, number of invasive foci, predictors, prognosis

## Abstract

Multiple invasive foci has been shown to increase the risk of lymph node metastasis (LNM) in early breast cancer, but its prognostic implication remains unknown. We aimed to identify the prognostic value of the number of invasive foci in ductal carcinoma in situ with minimal invasion of the breast (DCIS‐MI), and further establish a prognostic invasive lesion index (ILI). A total of 193 patients with DCIS‐MI (the invasive component was up to 10 mm in diameter) were included. Univariate and multivariate analysis (logistic regression) were used to evaluate the predictive value of the number of invasive foci in LNM. The Kaplan–Meier curve was used for survival analysis. More than five invasive foci was an independent predictor for LNM (OR, 2.67, 95% CI, 1.12–6.33, *P *=* *0.026), and associated with significantly shorter disease‐free survival (DFS) and overall survival (OS) compared with no more than five invasive foci (mean DFS 123.8 vs. 148.0 months, *P *=* *0.002; and mean OS 133.5 vs. 151.4 months, *P *=* *0.025). The ILI was established by the sum scores of the number of invasive foci and the invasive component size, having an optimal cut‐off point of 5.5 scores. The high‐ILI group (ILI >5 scores) had a higher incidence of LNM (23.6% vs. 6.9%) and worse prognosis than the low‐ILI group (ILI ≤5 scores). In conclusion, more than five invasive foci was an independent predictor for LNM and an unfavorable prognostic parameter. The ILI could potentially be used to predict survival prognosis in patients with DCIS‐MI.

## Introduction

Breast cancer is the most common malignant tumor and the second leading cause of death for females worldwide 1. The prognosis depends largely on the tumor size and lymph node status at the time of detection. Carcinomas with larger tumor size or lymph node metastasis (LNM) often have poor survival outcomes 2, 3.

Ductal carcinoma in situ associated with invasive carcinoma is considered to be an intermediate state between ductal carcinoma in situ and invasive ductal carcinoma 4, and is generally treated according to the guidelines for invasive disease. The definition of ductal carcinoma in situ with minimal invasion of the breast (DCIS‐MI) is still ambiguous. Generally, DCIS‐MI refers to invasive component up to 10 mm in diameter 5. Based on the TNM staging system of AJCC (7th edition), the tumor T stage of DCIS‐MI is T1mi, T1a, or T1b. As indicated by the National Comprehensive Cancer Network clinical practice guidelines 6, T1mi and T1a without LNM are not necessary to receive adjuvant chemotherapy. T1b without LNM is considered to receive adjuvant chemotherapy when patients have unfavorable prognostic features including intramammary angiolymphatic invasion, high nuclear grade, high histological grade, human epidermal growth factor receptor‐2 (HER‐2)‐positive status, or hormone receptor‐negative status.

Despite the vast majority of patients with DCIS‐MI have excellent survival prognosis, some of them are confirmed with LNM at the time of initial diagnosis 7, or subsequently develop metastatic disease after standard adjuvant therapies. This indicates that additional prognostic factors are needed so as to identify and properly treat higher risk patients even in this category of the tumors. Previous studies have demonstrated that carcinomas with multiple invasive foci may indicate a greater tumor burden, and thus have a stronger invasion and metastasis ability. Compared with those having one invasive focus, patients harboring more than one focus of microinvasion might have an increased risk of LNM, and probably worse prognosis 8, 9, 10.

Therefore, we presumed that multiple invasive foci is an unfavorable prognostic factor for DCIS‐MI. So far, this issue has not been thoroughly understood. Based on this premise, we performed this retrospective cohort study to identify the prognostic implication of the number of invasive foci in patients with DCIS‐MI, and further establish a prognostic invasive lesion index (ILI) based on the number of invasive foci.

## Materials and Methods

### Study population

With institutional review board approval, we first retrospectively investigated the records from a prospectively collected database maintained by Sun Yat‐sen University Cancer Center (SYSUCC), and selected those diagnosed with DCIS with coexisting minimal invasion, early invasion, satellite invasive foci, and microinvasion as potential eligible patients. Then, the pathologist (LM) reviewed again all the histological slides of the potential eligible patients. Only those with DCIS‐MI from January 2002 to December 2015 were eligible for inclusion. Other inclusion criteria included the following: (1) female; (2) the invasive component was up to 10 mm in diameter; and (3) the patients had received axillary lymph node dissection or sentinel lymph node biopsy so that the axillary lymph node status was definite. Patients were excluded if they: (1) received neoadjuvant chemotherapy; (2) had bilateral breast cancer; (3) had previous or coexisting cancers other than breast cancer; or (4) had confirmed metastasis. Written informed consent about researchable use of the clinical data was obtained from every participant prior to surgery. The conditions of the included patients were confirmed by routine tests and telephone counseling, and they were followed up until December 31, 2016 or the date of death.

### Data collection

Data on the number of invasive foci, the presence of lymphatic vascular invasion (LVI), the maximal diameter, estrogen receptor (ER), progesterone receptor (PR), HER‐2 status, and Ki67 of in situ component and invasive component were collected by a single senior pathologist (LM) at the SYSUCC. The clinical stages were classified according to the TNM staging system of AJCC (7th edition). The molecular subtypes were as follows: Luminal A (ER positive, PR positive, HER‐2 negative and Ki67 ≤ 14%), Luminal B (ER positive and HER‐2 positive or Ki67 > 14%), HER‐2 overexpressing (ER negative, PR negative, and HER‐2 positive), and triple‐negative breast cancer (ER negative, PR negative, and HER‐2 positive). HER‐2 positive was defined as immunohistochemical grade of 3+ or determined by fluorescence in situ hybridization as positive 11. In addition, other clinical data were also obtained from the database, including age at diagnosis, menstrual status, lymph node status, local or regional recurrence, distant metastasis and death events, the date of recurrence, metastasis, death, and last follow‐up.

### Statistical analysis

The disease‐free survival (DFS) was calculated from the date of diagnosis to the date of recurrence, metastasis, death, or last follow‐up. The overall survival (OS) was calculated from the date of diagnosis to the date of death or last follow‐up. Median and range were used to describe the continuous data. Numbers and percentages were used to describe the categorical data. The chi‐square test was considered appropriate to evaluate the ER, PR, and HER‐2 status and molecular subtypes between in situ and invasive components. Univariate and multivariate analysis (logistic regression) were responsible for evaluating the predictive value of the number of invasive foci on the risk of LNM. The independent factors associated with the risk of LNM were allocated with scores and the ILI was established based on the sum of the total scores. Receiver operating characteristic curve (ROC) was carried out to determine the optimal cut‐off point of the ILI and patients were stratified into the low‐ILI group and the high‐ILI group by the optimal cut‐off point. The chi‐square test was used to evaluate the differences in patients' characteristics between these two groups. A Kaplan–Meier curve was used for survival analysis, and differences between groups were assessed by log‐rank test. A two‐tailed *P* value of <0.05 was considered statistically significant. All of the statistical analysis was performed using SPSS, version 19.0 (SPSS Inc., Chicago, IL).

## Results

### Baseline characteristics

A total of 193 patients with DCIS‐MI were enrolled. The median follow‐up time was 46 months (range, 9–156 months), and the baseline clinicopathologic characteristics of the included patients are summarized in Table 1. The median age was 47 years (range, 22–87 years), and 17 (8.8%) patients were younger than 35 years. The median diameters of in situ and invasive components were 3 cm (range, 0.5–9 cm) and 2.5 mm (range, 0.1–10 mm), respectively. There were 59 (30.6%), 101 (52.3%), and 33 (17.1%) patients classified as T1mi, T1a, and T1b, respectively. Seventy‐one (36.8%) patients had one invasive focus and 72 (37.3%) had more than five invasive foci. Eight (4.1%) patients were confirmed to have LVI. During the follow‐up period, there were three local or regional recurrence, 11 distant metastasis and five death events.

**Table 1:** Baseline characteristics

Variable	All patients, *n* (%)
No. of patients	193 (100)
Age at diagnosis, years	
≤35	17 (8.8)
>35	176 (91.2)
Menopause	
No	120 (62.2)
Yes	73 (37.8)
In situ component size, cm	
≤2	68 (35.2)
2–5	103 (53.4)
>5	22 (11.4)
Number of invasive foci	
1	71 (36.8)
2	23 (11.9)
3	16 (8.3)
4	6 (3.1)
5	5 (2.6)
>5	72 (37.3)
T stage of invasive component	
T1mic	59 (30.6)
T1a	101 (52.3)
T1b	33 (17.1)
Presence of LVI	
No	185 (95.9)
Yes	8 (4.1)
Local or regional recurrence	
No	190 (98.4)
Yes	3 (1.6)
Distant metastasis	
No	182 (94.3)
Yes	11 (5.7)
Death	
No	188 (97.4)
Yes	5 (2.6)

LVI, lymphatic vascular invasion.

### Molecular marker status of in situ and invasive components

Table 2 displays the molecular marker status of in situ and invasive components. For the in situ component, 104 (53.9%), 98 (50.8%), and 90 (46.6%) patients were ER positive, PR positive, and HER‐2 positive, respectively. Luminal B was the most commonly seen subtype, accounting for 31.6% (61/193). The percentages of luminal A, HER‐2 overexpressing ,and triple‐negative subtypes were 19.2%, 25.4%, and 9.3%, respectively. With regard to the invasive component, 99 (51.3%), 92 (47.7%), and 81 (42.0%) patients were ER positive, PR positive, and HER‐2 positive, respectively. The percentages of patients with luminal A, Luminal B, HER‐2 overexpressing and triple‐negative subtypes were similar with those of the in situ component (16.1%, 31.6%, 24.4%, and 11.4%, respectively).

**Table 2:** Molecular marker status of in situ and invasive components

	In situ component	Invasive component	*P*
ER			0.610
Positive	104 (53.9)	99 (51.3)	
Negative	89 (46.1)	94 (48.7)	
PR			0.541
Positive	98 (50.8)	92 (47.7)	
Negative	95 (49.2)	101 (52.3)	
HER‐2			0.637
Positive	90 (46.6)	81 (42.0)	
Negative	75 (38.9)	80 (41.5)	
Unknown	28 (14.5)	32 (16.6)	
Molecular subtype			0.872
Luminal A	37 (19.2)	31 (16.1)	
Luminal B	61 (31.6)	61 (31.6)	
HER‐2 overexpressing	49 (25.4)	47 (24.4)	
Triple negative	18 (9.3)	22 (11.4)	
Unknown	28 (14.5)	32 (16.6)	
ER concordance			
Yes	180 (93.3)
No	13 (6.7)
PR concordance			
Yes	179 (92.7)
No	14 (7.3)
HER‐2 concordance			
Yes	170 (88.1)
No	23 (11.9)

ER, estrogen receptor; PR, progesterone receptor; HER‐2, human epidermal growth factor receptor‐2.

Of note, most patients had concordant ER, PR, and HER‐2 status between in situ and invasive components, but 13 (6.7%), 14 (7.3%), and 23 (11.9%) patients presented discordant ER, PR, and HER‐2 statuses, respectively. According to the chi‐square test, there was no statistically significant difference in the ER, PR, and HER‐2 status and percentages of molecular subtypes between in situ and invasive components (all *P *>* *0.05).

### The predictive value of the number of invasive foci in LNM

The overall incidence of LNM was 16.1% (31/193). For patients with more than five invasive foci, they had a higher incidence of 27.8% than those with no more than five invasive foci. Otherwise, for patients with an in situ component >5 cm in size, an invasive component >5 mm in size, or the presence of LVI, they also had a higher incidence of 27.3%, 36.4%, or 50.0%, respectively. The result of the univariate logistic regression demonstrated that a statistically significant association was found between the number of invasive foci and risk of LNM, as well as between the invasive component size, the presence of LVI, and risk of LNM. Compared to patients with no more than five invasive foci, the risk of LNM was 3.8‐fold (95% confidence interval [CI], 1.72–8.61, *P *=* *0.001) higher in patients with more than five invasive foci. No statistical significance was observed between ER, PR, and HER‐2 status and molecular subtypes of invasive component and risk of LNM (all *P *>* *0.05). The number of invasive foci, invasive component size, and the presence of LVI were further assessed by the multivariate logistic regression analysis and the results demonstrated that, the number of invasive foci could still predict the risk of LNM with an odds ratio (OR) of 2.67 (95% CI, 1.32–6.33, *P *=* *0.026) (shown in Table 3).

**Table 3:** Logistic regression of factors associated with risk of lymph node metastasis

Variable	Incidence of LNM, %	Univariate analysis		Multivariate analysis^a^	
		OR (95% CI)	*P*	OR (95% CI)	*P*
In situ component size, cm					
≤5	14.6	1		–	
>5	27.3	2.19 (0.78–6.13)	0.136	–	–
Number of invasive foci					
≤5	9.1	1		1	
>5	27.8	3.85 (1.72–8.61)	0.001	2.67 (1.32–6.33)	0.026
Invasive component size, mm					
≤5	11.9	1		1	
>5	36.4	4.24 (1.80–9.98)	0.001	3.30 (1.33–8.19)	0.010
Presence of LVI					
No	14.6	1		1	
Yes	50.0	5.85 (1.38–24.82)	0.017	3.85 (0.81–18.23)	0.089
ER of invasive component					
Negative	16.0	1		–	
Positive	16.2	1.02 (0.47–2.19)	0.969	–	–
PR of invasive component					
Negative	14.9	1		–	
Positive	17.4	1.21 (0.56–2.60)	0.632	–	–
HER‐2 of invasive component					
Negative	18.8	1		–	
Positive	14.8	0.75 (0.33–1.73)	0.505	–	–
Unknown	12.5	0.62 (0.19–2.03)	0.429	–	–
Molecular subtype of invasive component					
Luminal A	19.4	1		–	
Luminal B	18.0	0.92 (0.30–2.77)	0.877	–	–
Her‐2 overexpressing	12.8	0.61 (0.18–2.10)	0.433	–	–
Triple negative	18.1	0.93 (0.15–2.36)	0.914	–	–

LNM, lymph node metastasis; OR, odds ratio; CI, confidence interval; LVI, lymphatic vascular invasion; ER, estrogen receptor, PR, progesterone receptor; HER‐2, human epidermal growth factor receptor‐2.

a Adjusted for the number of invasive foci, invasive component size, and the presence of LVI.

### The prognostic implication of the number of invasive foci in survival outcomes

**Figure 1:**
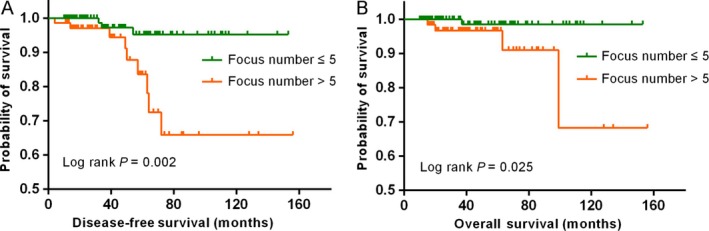
Survival analysis based on the number of invasive foci. Disease‐free survival (A) and overall survival (B) for patients with more than five invasive foci versus no more than five invasive foci.

To further evaluate the prognostic implication of the number of invasive foci, a Kaplan–Meier curve was constructed (shown in Fig. 1). The mean DFS of patients with more than five invasive foci was 123.8 months (95% CI, 105.0‐142.7), which was significantly shorter than that of patients with no more than five invasive foci (mean DFS, 148.0 months; 95% CI, 142.4–153.6; *P *=* *0.002). Similarly, patients with more than five invasive foci had significantly shorter mean OS than those with no more than five foci (mean OS, 133.5 months; 95% CI, 109.6–157.8; and mean OS, 151.4 months; 95% CI, 148.2–154.5, respectively; *P *=* *0.025).

### The establishment and prognostic implication of ILI

**Figure 2:**
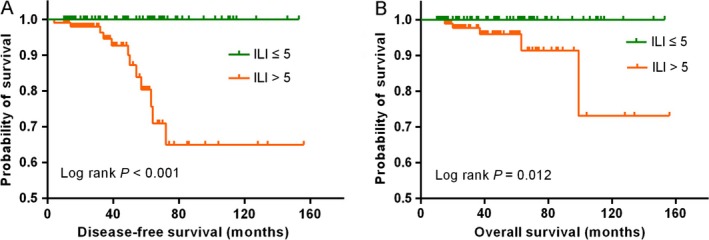
Survival analysis based on the invasive lesion index. Disease‐free survival (A) and overall survival (B) for patients in high‐ILI group versus patients in low‐ILI group.

The criteria for establishing ILI were as follows: 1, 2, 3, 4, 5, and >5 invasive foci were allocated 1, 2, 3, 4, 5, and 6 scores, respectively; invasive component ≤1 mm, 1–2 mm, 2–3 mm, 3–4 mm, 4–5 mm, and >5 mm in size were allocated 1, 2, 3, 4, 5, and 6 scores, respectively. The ILI was established by the sum scores of the number of invasive foci and the invasive component size, so the total scores of ILI ranged from 2 to 12. As shown by the results of ROC (shown in Fig. S1), the area under the curve for ILI in evaluating DFS was 0.798 (95% CI, 0.734–0.852, *P* < 0.001) with the optimal cut‐off point of 5.5 scores, and the area under the curve for ILI in evaluating OS was 0.690 (95% CI, 0.620–0.755, *P *=* *0.006) with the same optimal cut‐off point of 5.5 scores. Based on the optimal cut‐off point, patients were stratified into two groups: low‐ILI group with ILI ≤5 scores and high‐ILI group with ILI > 5 scores. Table 4 described the clinicopathologic features stratified by the ILI groups. Eighty‐seven (45.1%) patients were allocated into the low‐ILI group and 106 (54.9%) into the high‐ILI group. Patients in the high‐ILI group had a higher incidence of LNM than patients in the low‐ILI group (23.6% vs. 6.9%, respectively, *P *=* *0.002). No statistical significance was observed in the age at diagnosis, menstrual status, ER, PR, and HER‐2 status of the invasive component between the high‐ and low‐ILI groups. According to the Kaplan–Meier analysis, patients in the high‐ILI group had significantly shorter DFS and OS compared with patients in the low‐ILI groups (*P *<* *0.001, Fig. 2A and *P* = 0.012, Fig. 2B, respectively). In the subgroup of patients without LNM, those in high‐ILI group still had significant shorter DFS (shown in Fig. S3).

**Table 4:** The clinicopathologic features stratified by the ILI groups

Variable	Low‐ILI, *n* (%)	High‐ILI, *n* (%)	*P*
No. of patients	87	106	
Age at diagnosis, years			0.864
≤35	8 (9.2)	9 (8.5)	
>35	79 (90.8)	97 (91.5)	
Menopause			0.222
No	50 (57.5)	70 (66.0)	
Yes	37 (42.5)	36 (34.0)	
In situ component size, cm			0.045
≤2	38 (43.7)	30 (28.3)	
2–5	38 (43.7)	65 (61.3)	
>5	11 (12.6)	11 (10.4)	
Number of invasive foci			<0.001
1	53 (60.9)	18 (17.0)	
2	20 (23.0)	3 (2.8)	
3	12 (13.8)	4 (3.8)	
4	2 (2.3)	4 (3.8)	
5	0	5 (4.7)	
>5	0	72 (67.9)	
T stage of invasive component			<0.001
T1mic	45 (51.7)	14 (13.2)	
T1a	42 (48.3)	59 (55.7)	
T1b	0	33 (31.1)	
Presence of LVI			0.075
No	86 (98.9)	99 (93.4)	
Yes	1 (1.1)	7 (6.6)	
LNM			0.002
No	81 (93.1)	81 (76.4)	
Yes	6 (6.9)	25 (23.6)	
ER of invasive component			0.856
Negative	43 (49.4)	51 (48.1)	
Positive	44 (50.6)	55 (51.9)	
PR of invasive component			0.878
Negative	45 (51.7)	56 (52.8)	
Positive	42 (48.3)	50 (47.2)	
HER‐2 of invasive component			0.175
Negative	32 (36.8)	48 (45.3)	
Positive	36 (41.4)	45 (42.4)	
Unknown	19 (21.8)	13 (12.3)	

ILI, invasive lesion index; LNM, lymph node metastasis; ER, estrogen receptor; PR, progesterone receptor; HER‐2, human epidermal growth factor receptor‐2.

## Discussion

In this study, we retrospectively reviewed the tumor specimen slides of 193 patients diagnosed with DCIS‐MI to identify the prognostic value of the number of invasive foci, and to establish a novel prognostic index. Our main findings were as follows: (1) more than five invasive foci was an independent predictor for LNM and an unfavorable prognostic factor; and (2) the ILI established by the number of invasive foci and the invasive component size could potentially be used to evaluate the risk of LNM and survival prognosis.

According to NCCN guidelines, generally, DCIS‐MI gets the same treatments as pure small invasive breast cancer (pure T1a, T1b), depending mainly on the largest diameter of its invasion foci. However, based on our observation in the clinical practice, DCIS‐MI is more often present with multiple foci of invasion, while pure IDC is always single lesion. As shown in our results, 71 (36.8%) patients had one invasive focus and 72 (37.3%) had more than five invasive foci. Thus, we chose this specific group of patients to study the prognostic effect of multiple foci of invasion. In this study, we considered well‐defined invasive foci to be individual foci when they were separated from each other by tissue that did not have invasive structures without respect to the distance between foci or the quadrant localization of the foci. The largest invasive focus diameter was used to approximate the tumor size for staging purpose according to the TNM staging system of AJCC (7th edition).

As proved by the previous studies, younger age, increasing tumor size, poor histological grade, and the presence of LVI are the most important predictive parameters for LNM in early breast cancer 12, 13, 14. In this study, more than five invasive foci was an independent risk factor for LNM with an OR of 2.67 (*P *=* *0.026). Therefore, it could be an additional predictor for LNM in this tumor category. This conclusion is consistent with previous findings 15, 16, 17. In a study of 301 consecutive cases of 1–14 mm invasive breast carcinomas, Tibor and his colleagues found that mutifocality of the invasive component was associated with a substantially elevated risk of vascular invasion and LNM 18. That study did not further explore the effect of the multifocality of the invasive component on survival prognosis. Theoretically, the invasive foci might result from the intramammary tumor spread and thus indicate stronger invasion and metastasis ability, because intramammary spread of the tumor may be the initial step in generating LNM 18. In addition, the Kaplan–Meier curve analysis in this study demonstrated that more than five invasive foci was also an unfavorable prognostic factor for patients with DCIS‐MI.

Currently, the prognosis assessment of breast cancer is mainly based on the tumor size, lymph node status, and molecular marker status. Recently, the 21‐gene expression assay has been added in the TNM staging system of AJCC (8th edition) as one of the prognostic indicators 19. However, the 21‐gene expression assay fails to predict the benefit of adjuvant chemotherapy for patients who are classified as having intermediate risk of recurrence. As shown in our results, patients in the high‐ILI group had a significantly higher incidence of LNM and shorter DFS and OS than those in the low‐ILI group. In addition, aside from the number of invasive foci and invasive component size, the molecular biomarkers (ER, PR, HER‐2) present no prognostic effect on LNM. Thus, ILI is a promising prognostic factor especially in the issue of DCIS‐MI. According to the present NCCN guideline, generally DCIS‐MI gets the same treatments as pure small invasive breast cancer (pure T1a, T1b), depending mainly on the largest diameter of its invasion foci. For example, DCIS with five invasive foci (all <5 mm) and no LNM is staged as pT1aN0M0 and would receive no adjuvant chemotherapy. However, our results showed that it would get a high‐ILI score and poor prognosis, which could help in clinical decision‐making for physicians (such as adding chemotherapy).

Some limitations of this study were characterized. First, the data were retrospectively collected from a single center, although the included patients were consecutive and this is unlikely to influence the ascertainment of LNM. Second, the sample size might be insufficient because the incidence of DCIS‐MI was very low in the clinical practice. Third, the prognostic value of more than five invasive foci and the ILI was not further confirmed by the multivariate Cox regression analysis because of the small sample size and the short follow‐up time; thus, the results might be distorted by confounders. Therefore, further prospective studies of larger cohorts with longer follow‐up time are warranted to confirm the results of our study.

In summary, this study demonstrated that more than five invasive foci was an independent predictor for LNM and an unfavorable prognostic parameter for patients with DCIS‐MI. Furthermore, the ILI could potentially be an important prognostic factor and assist clinicians in deciding personalized treatment strategies.

## Conflict of Interest

The authors declare that they have no conflict of interest.

## Supporting information


**Figure S1.** The receiver operating characteristic curves of the invasive lesion index.

**Figure S2.** Survival analysis based on the number of invasive foci stratified by lymph node status.

**Figure S3.** Survival analysis based on invasive lesion index (ILI) stratified by lymph node status.
