# Supplementary figures and images for: Application of a novel prognostic invasive lesion index in ductal carcinoma in situ with minimal invasion of the breast
Source: Cancer Med. 2017 Oct 4;6(11):2489–96. doi: 10.1002/cam4.1175 (PMC5673919; doi:10.1002/cam4.1175)

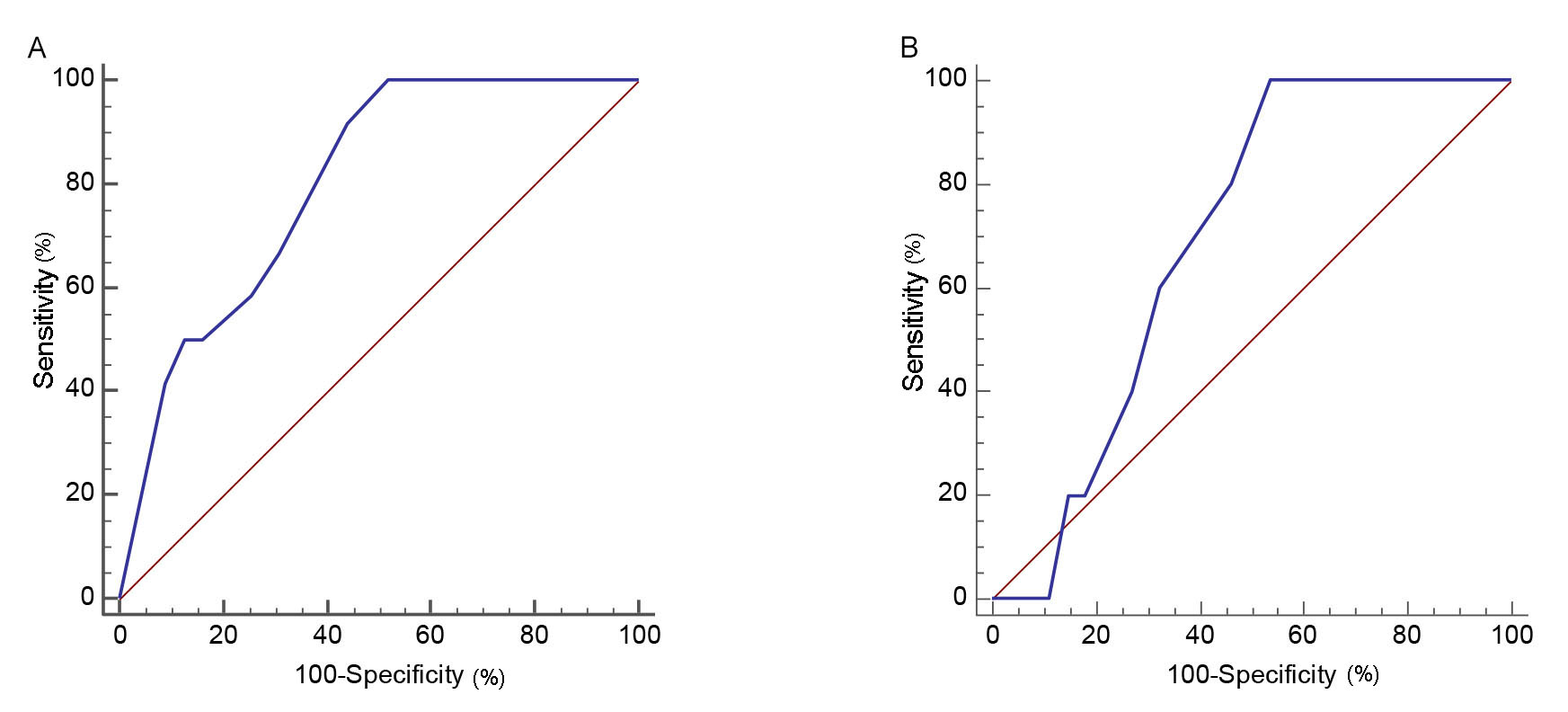

Supplement: Supplementary file 1 — Figure S1. The receiver operating characteristic curves of the invasive lesion index. [file CAM4-6-2489-s001.tif]

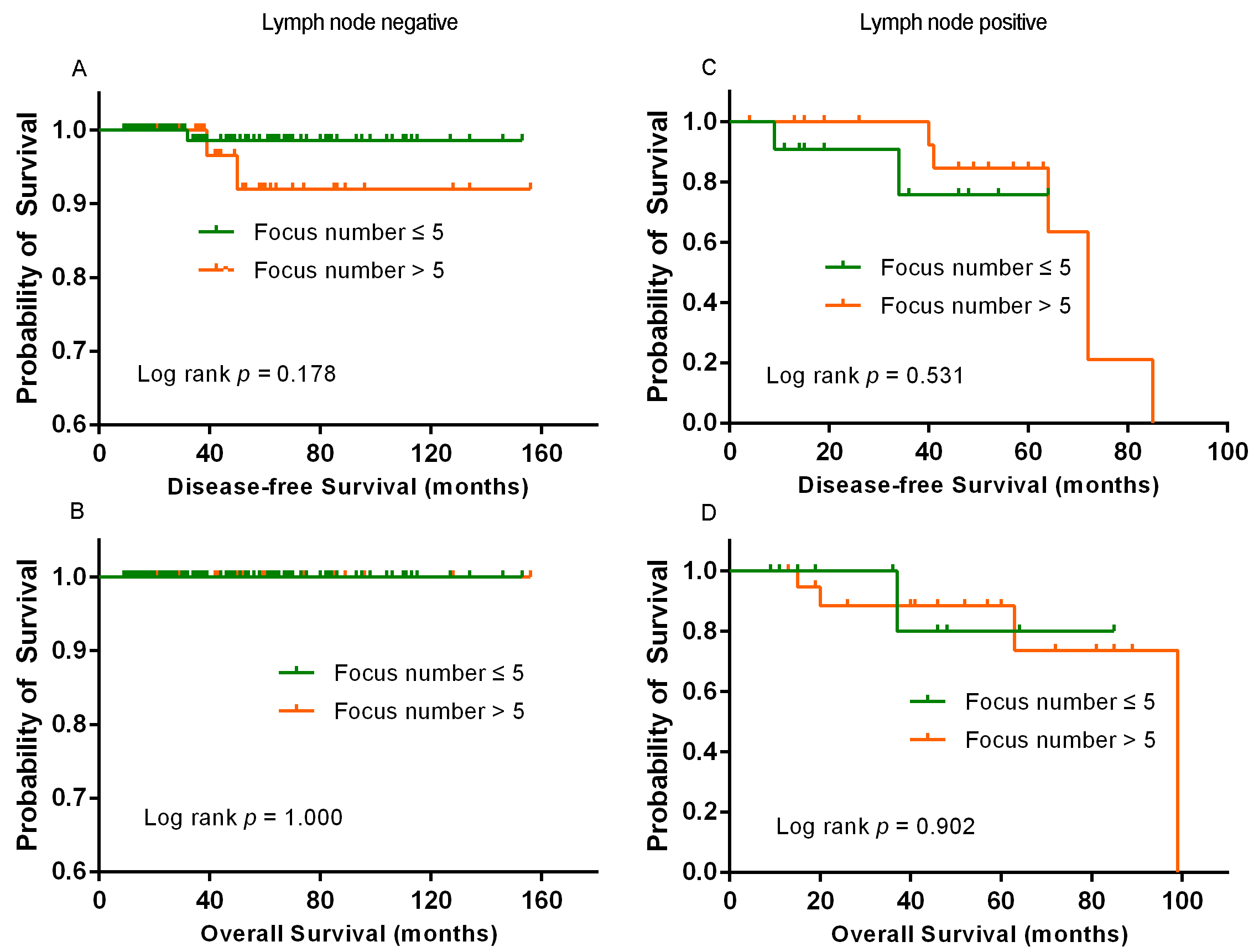

Supplement: Supplementary file 2 — Figure S2. Survival analysis based on the number of invasive foci stratified by lymph node status. [file CAM4-6-2489-s002.tif]

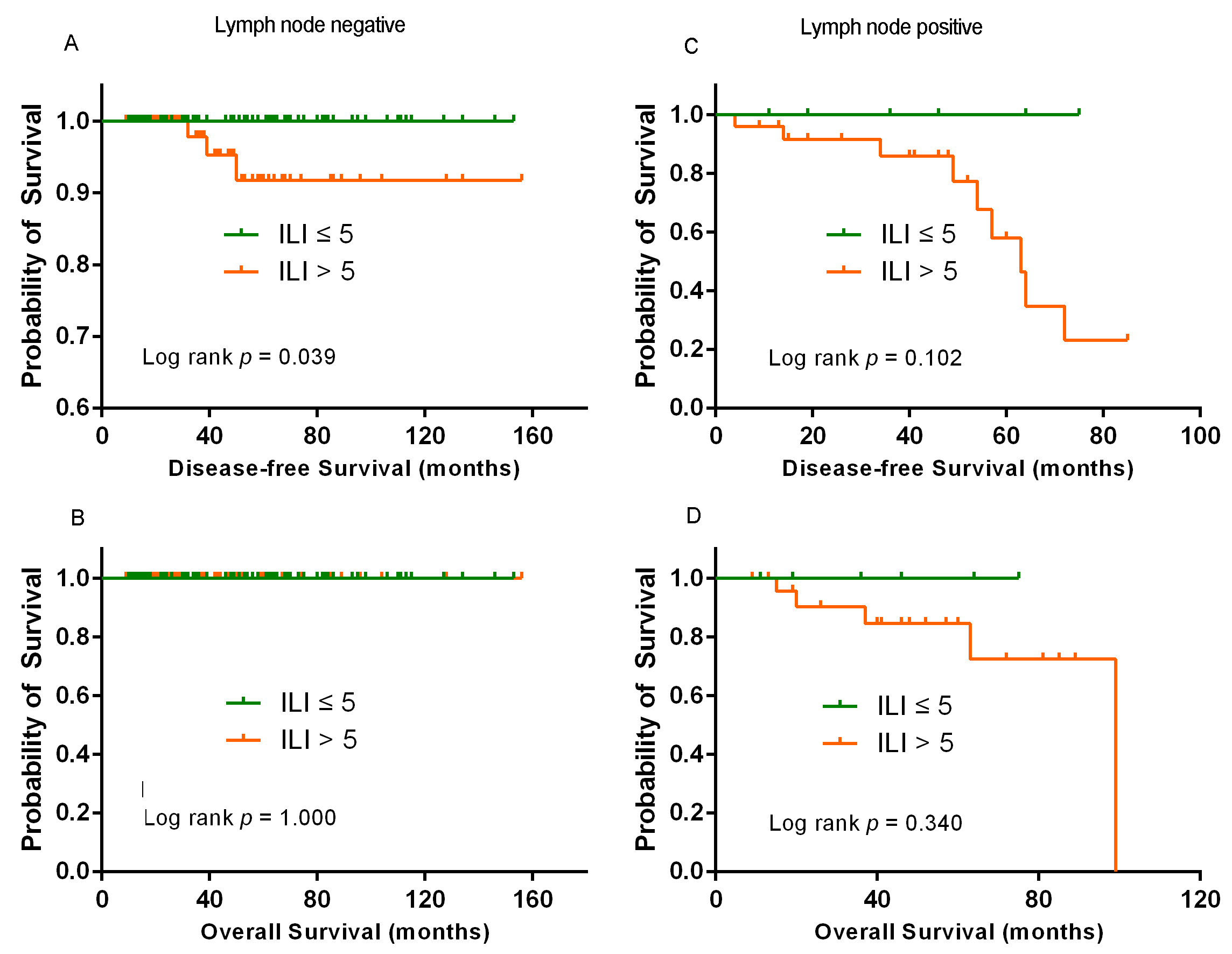

Supplement: Supplementary file 3 — Figure S3. Survival analysis based on invasive lesion index (ILI) stratified by lymph node status. [file CAM4-6-2489-s003.tif]
